# Dietary oleuropein inhibits tumor angiogenesis and lymphangiogenesis in the B16F10 melanoma allograft model: a mechanism for the suppression of high-fat diet-induced solid tumor growth and lymph node metastasis

**DOI:** 10.18632/oncotarget.16757

**Published:** 2017-03-31

**Authors:** Hyerim Song, Do Young Lim, Jae In Jung, Han Jin Cho, So Young Park, Gyoo Taik Kwon, Young-Hee Kang, Ki Won Lee, Myung-Sook Choi, Jung Han Yoon Park

**Affiliations:** ^1^ Department of Food Science and Nutrition, Hallym University, Chuncheon 24252, Republic of Korea; ^2^ The Hormel Institute, University of Minnesota, Austin, MN 55912, USA; ^3^ Division of Bio-Imaging, Chuncheon Center, Korea Basic Science Institute, Chuncheon 24341, Republic of Korea; ^4^ WCU Biomodulation Major, Department of Agricultural Biotechnology and Center for Food and Bioconvergence, Seoul National University, Seoul 08826, Republic of Korea; ^5^ Advanced Institutes of Convergence Technology, Seoul National University, Suwon 16229, Republic of Korea; ^6^ Berry and Biofood Research Institute, Jeonbuk 56417, Republic of Korea; ^7^ Research Institute of Agriculture and Life Science, Seoul National University, Seoul 08826, Republic of Korea; ^8^ Department of Food Science and Nutrition, Kyungpook National University, Daegu 41566, Republic of Korea

**Keywords:** oleuropein, melanoma, metastasis, angiogenesis, lymphangiogenesis

## Abstract

Previously, we reported that high-fat-diet (HFD)-induced obesity stimulates melanoma progression in the B16F10 allograft model. In this study, we examined whether oleuropein (OL), the most abundant phenolic compound in olives, inhibits HFD-induced melanoma progression. Four-week-old male C57BL/6N mice were fed a HFD-diet with or without OL. After 16 weeks of feeding, B16F10-luc cells were subcutaneously injected and the primary tumor was resected 3 weeks later. OL suppressed HFD-induced solid tumor growth. In the tumor tissues, OL reduced HFD-induced expression of angiogenesis (CD31, VE-cadherin, VEGF-A, and VEGFR2), lymphangiogenesis (LYVE-1, VEGF-C, VEGF-D, and VEGFR3), and hypoxia (HIF-1α and GLUT-1) markers as well as HFD-induced increases in lipid vacuoles and M2 macrophages (MΦs). All animals were euthanized 2.5 weeks after tumor resection. OL suppressed HFD-induced increases in lymph node (LN) metastasis; expression of VEGF-A, VEGF-C, and VEGF-D in the LN; and M2-MΦs and the size of adipocytes in adipose tissues surrounding LNs. Co-culture results revealed that the crosstalk between B16F10s, M2-MΦs, and differentiated 3T3-L1 cells under hypoxic conditions increased the secretion of VEGF-A and -D, which stimulated tube formation and migration of endothelial cells (HUVECs) and lymphatic endothelial cells (LEC), respectively. Additionally, OL directly inhibited the differentiation of 3T3-L1 preadipocytes and tube formation by HUVECs and LECs. The overall results indicated that dietary OL inhibits lipid and M2-MΦ accumulation in HFD-fed mice, which contributes to decreases in VEGF secretion, thereby leading to inhibition of angiogenesis and lymphangiogenesis.

## INTRODUCTION

Epidemiological studies indicate that overweight and obesity are associated with increased risks of developing several cancers, including melanoma [1–3]. It has been reported that 15–20% of all cancer deaths in the Unites States are due to overweight and obesity [4]. Additionally, our previous report demonstrated that high-fat diet (HFD)-induced obesity stimulates tumor growth and lymph node (LN) metastasis of B16F10 melanoma cells in C57BL/6 mice [5].

Metastasis is a leading cause of mortality in cancer patients. Metastasis, the spread of malignant cells from a primary tumor site to different sites of the same organ or to distant organs, is a multi-step process that involves cell migration, adhesion, invasion, angiogenesis, and lymphangiogenesis (reviewed in [6–10]) Metastasis occurs through blood and lymphatic vessels [8, 10]. Tumor angiogenesis is a major feature of tumor growth and metastasis, and includes endothelial cell proliferation and migration, tube formation, degradation of extracellular matrix, and sprouting of new capillaries (reviewed in [11]). Lymphangiogenesis also requires the coordination of several cellular events, including proliferation, sprouting, migration and tube formation, similar to events related to angiogenesis (reviewed in [10]). Vascular endothelial growth factor (VEGF) is a major pro-angiogenic cytokine, which comprises several isotypes, including VEGF-A, VEGF-B, VEGF-C and VEGF-D, as splice variant isoforms [12]. VEGF-A is a well-established key regulator of vasculogenesis and angiogenesis. VEGF-C and VEGF-D are associated with tumor lymphangiogenesis and metastasis. Furthermore, VEGF-C and VEGF-D produced by tumor cells induce sprouting of lymphatic capillaries and dilation of the draining peritumoral lymphatic vessels [13, 14]. In the tumor microenvironment, VEGFs bind to VEGF receptor (VEGFR)s on neighboring endothelial cells and promote the growth and development of new blood vessels [15].

The tumor microenvironment is composed of proliferating tumor cells, blood vessels, infiltrating inflammatory cells, adipocytes and a variety of associated tissue cells. Many studies have indicated that tumor-associated macrophages (TAMs) exhibit several M2-associated protumoral functions, including promotion of tumor growth, invasion, metastasis, and angiogenesis (reviewed in [16]).

Tumor-associated adipocytes are also a critical component of the tumor microenvironment. Nieman *et al*. [17] reported adipocytes as the major mediators of ovarian cancer metastasis to the omentum. In addition, Rahimi *et al*. showed that 3T3-L1 adipocytes stimulate murine mammary carcinoma cell growth by secreting hepatocyte growth factor [18]. Several studies indicate that obese mice exhibit increased melanoma growth *in vivo* [5, 19, 20]. Furthermore, conditioned media of 3T3-L1 cells and adipose tissues stimulate B16F10 melanoma cell proliferation, migration and invasion, and reduce apoptosis *in vitro* and *ex vivo* [21].

In the tumor microenvironment, areas of hypoxia are enlarged due to rapid increases in tumor mass. Hypoxia is a fundamental stimulus of angiogenesis, which is essential for malignant tumor growth and wound healing [22, 23]. The hypoxia response is mediated largely by hypoxia-inducible factor (HIF)s. HIF-1α accumulates under hypoxic conditions and plays an important role in immune and inflammatory responses. Previous studies have shown that HIF-1α is activated in the tumor tissues of mice fed a HFD [5, 24], and has a role in the regulation of important features of angiogenesis [25, 26]. Hypoxic conditions upregulate VEGF [27] due to increases in HIF-1 and VEGF mRNA levels [28].

Oleuropein (OL) is the most abundant phenolic compound in olives, and has antioxidant, antimicrobial, and antiobesity activities [29–31]. Additionally, oral (1% OL in drinking water) OL exerts anticancer effects in mice that developed spontaneous tumors and inhibits the proliferation and migration of several tumor cell lines *in vitro* [32]. However, the effects of OL on obesity-induced tumor progression have not been reported.

In the present study, we showed that dietary OL suppresses HFD-stimulated solid tumor growth and LN metastasis using the B16F10 allograft model. In addition to its direct effect on endothelial cells, OL decreases major factors of angiogenesis (VEGF-A) and lymphangiogenesis (VEGF-D) by decreasing the numbers of adipocytes and M2-MΦs in these mice. Our results also indicated that the crosstalk between adipocytes, M2-MΦs and B16F10 melanoma cells under hypoxic conditions is important for angiogenesis and lymphangiogenesis, and is blocked by OL.

## RESULTS

### Dietary OL inhibits HFD-stimulated solid tumor growth and LN metastasis

**Figure 1:**
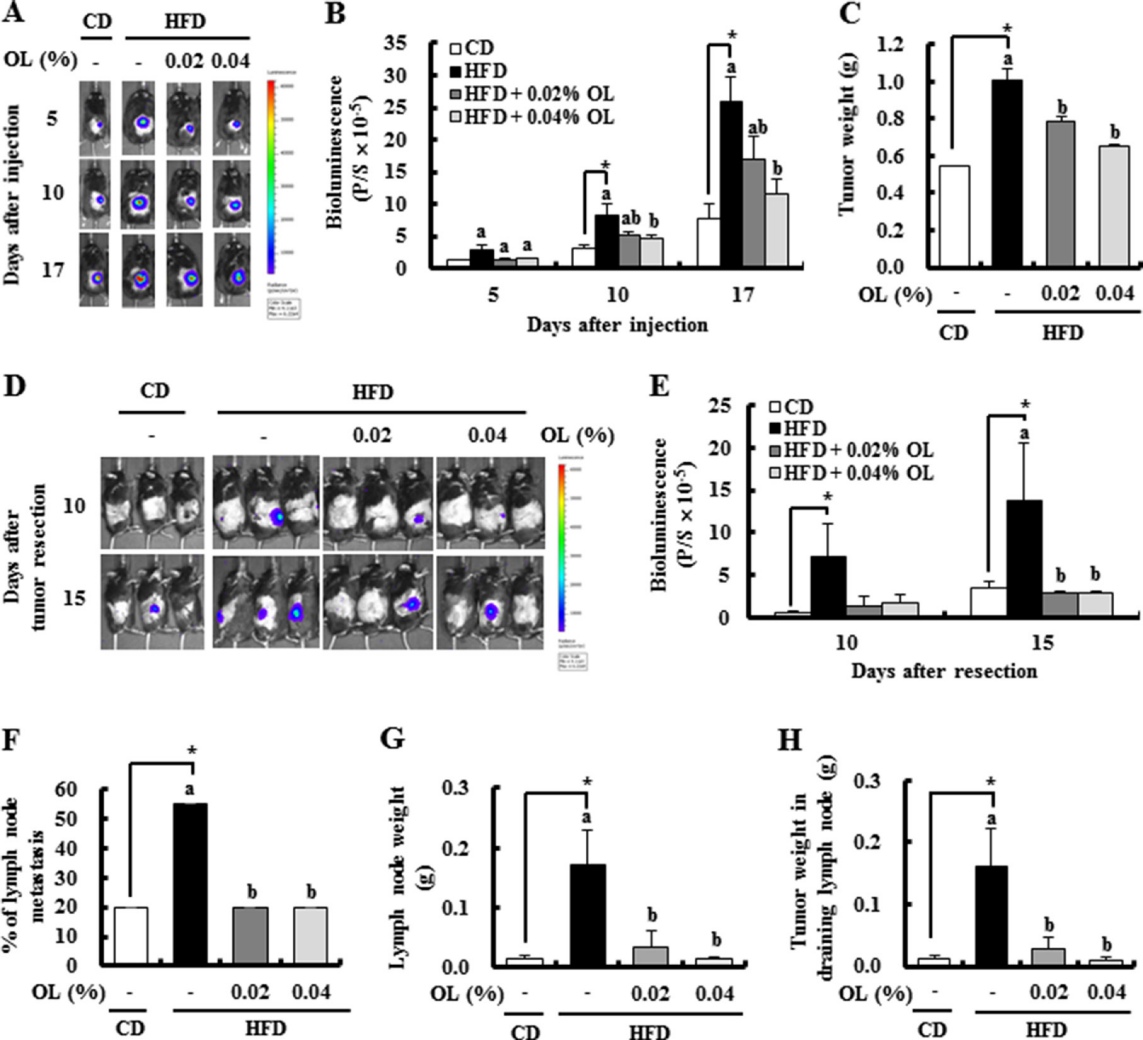
OL suppresses HFD-stimulated tumor growth and lymph node (LN) metastasis in C57BL/6 mice injected with B16F10 cells (**A**, **B**) Bioluminescence imaging (BLI) was conducted at 5, 10, and 17 days after B16F10-luc cell injection. (A) Representative images of bioluminescent signals. (B) Quantitative analysis of luciferase signals. (**C**) The primary tumor was resected 3 weeks after injection of B16F10 cells and weighed. (**D**, **E**) After resecting the tumors, bioluminescent signals from the draining LNs were monitored at 10 and 15 days after resection. (D) Representative images of luciferase signals. (E) Quantitative analysis of luciferase signals. (F-H) The LNs were removed 17 days after tumor resection. (**F**) The incidence of metastasis to LNs. (**G**) The draining LN weight. (**H**) The tumor weights in the draining LN. Each bar represents the mean ± SEM (*n* = 20). *Significantly different from the CD group, *P* < 0.05. Means without a common letter differ among the three HFD groups, *P* < 0.05.

To determine whether dietary OL suppresses HFD-induced solid tumor growth *in vivo*, 4-week-old C57BL/6N mice were fed a control diet (CD) or HFD containing either 0.02% or 0.04% OL (Supplementary Figure 1). After 16 weeks of feeding, B16F10-luc cells were subcutaneously injected into the right rear flanks of the mice. After tumor cell injection, bioluminescence imaging (BLI) of the tumor region showed that tumor growth was markedly greater in HFD-fed mice than in CD-fed mice and was significantly decreased in the HFD + 0.04% OL-fed mice, as compared to the HFD-fed group (Figure 1A and 1B). At the time of tumor resection, tumor weights were significantly higher in the HFD group than the CD group, and dietary OL (0.02–0.04%) significantly decreased tumor weights (Figure 1C).

Dietary OL suppressed HFD-induced body weight gain. As compared to the HFD group, the HFD + 0.04% OL group showed a significantly lower mean body weight at 5–19 weeks after diet feeding; whereas, no significant difference in the mean body weight at 21 weeks after diet feeding (Supplementary Figure 2A). In addition, body composition was determined at 14 weeks after diet feeding. Dietary supplementation of OL resulted in significant suppression of the HFD-induced body fat mass (Supplementary Figure 2B and 2C). To determine whether OL inhibits adipocyte differentiation, its effect on 3T3-L1 differentiation was examined. Oil Red O staining was performed to evaluate lipid accumulation. OL at 10 μmol/L significantly decreased lipid accumulation in 3T3-L1 cells (Supplementary Figure 3A). However, OL treatment did not reduce the viability of 3T3-L1 cells at 2.5–10 μmol/L (Supplementary Figure 3B). Fasting blood glucose and plasma insulin levels were significantly higher in the HFD compared with the CD group, and reduced by 0.02–0.04% OL supplementation. To assess fasting insulin sensitivity, the homeostatic model assessment (HOMA)-insulin resistance (IR) was calculated. The HOMA-IR analysis indicated significant increases in insulin resistance in the HFD group compared to the CD group. Dietary OL significantly reduced the HOMA-IR values in HFD-fed mice (Supplementary Figure 2D–2F).

To determine the role of OL in B16F10 melanoma metastasis, we utilized the *in vivo* BLI system. Luminescence signals were detected at 10 and 15 days after tumor resection in the lymph node area, and dietary OL significantly decreased HFD-induced LN metastasis (Figure 1D and 1E). At the time of sacrifice, the draining LNs were dissected and the incidence of LN metastasis was estimated. The HFD group showed an increase in the incidence of metastasis (4 of 20 mice in the CD group; and 11/20 in the HFD group) (Figure 1F), as well as the weights of the draining LNs (Figure 1G), and tumors in the LNs (Figure 1H). OL suppressed the HFD-induced increase in the incidence of LN metastasis (4/20 mice in the HFD + 0.02% OL group; and 4/20 mice in the HFD + 0.04% OL group) (Figure 1F), the weights of draining LNs (Figure 1G), and tumors in the LNs (Figure 1H).

### Dietary OL suppresses HFD-stimulated lipid accumulation, cell proliferation, angiogenesis, and lymphangiogenesis and HFD-induced decreases in apoptosis in B16F10 solid tumors

**Figure 2:**
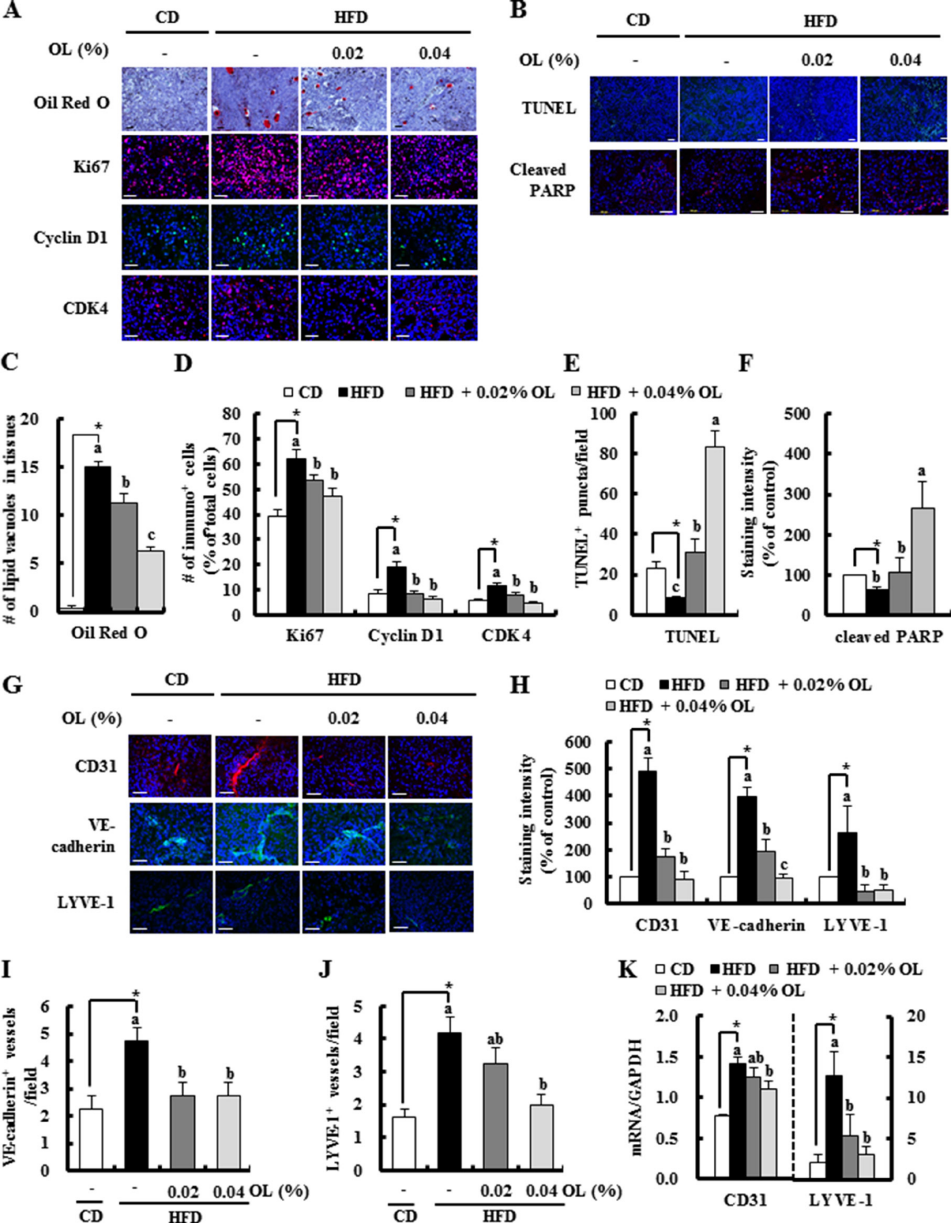
OL suppresses HFD-stimulated accumulation of lipid droplets, cell proliferation, angiogenesis, and lymphangiogenesis and HFD-induced decreases in apoptosis in B16F10 solid tumors Tumor sections were stained with Oil Red O and hematoxylin, indicated antibodies or TUNEL. (**A**, **B**, **G**) Representative Oil Red O-stained and immunofluorescence images are shown. (**C**) The lipid vacuoles were counted. Each bar represents the mean ± SEM (*n* = 5). (**D**) Ki67, Cyclin D1, and CDK4-positive cells, (**E**) The numbers of TUNEL-positive puncta, (**F**) staining intensity of cleaved PARP, and (**H**) staining intensity of CD31, VE-cadherin, and LYVE-1 were quantified. (**I**) VE-cadherin- and (**J**) LYVE-1-positive microvessels were quantified. Each bar represents the mean ± SEM (*n* = 5–9). (**K**) The levels of CD31 and LYVE-1 gene expression in tumors, as determined by real-time RT-PCR (mean ± SEM, *n* = 8–10). *Significantly different from the CD group, *P* < 0.05. Means without a common letter differ among the three HFD groups, *P* < 0.05.

Because dietary supplementation of OL resulted in significant suppression of the HFD-induced body fat mass, we examined whether HFD and dietary OL affect lipid accumulation in tumor tissues. Oil Red O staining showed that tumor tissues of the HFD group had excessive lipid vacuoles, which were decreased by OL supplementation (Figure 2A and 2C). Because dietary OL inhibited HFD-induced B16F10 solid tumor growth, we next examined the effect of OL on the levels of proteins involved in cell proliferation and apoptosis in B16F10 tumors. Immunofluorescence (IF) staining revealed that OL inhibited HFD-induced increases in the expression of Ki67, cyclin D1, and cyclin-dependent kinase (CDK)4 (Figure 2A and 2D). HFD feeding decreased the number of terminal deoxynucleotidyl transferase-mediated dUTP nick end labeled (TUNEL)-positive puncta and the levels of cleaved PARP. OL supplementation markedly increased the number of TUNEL-positive puncta and cleaved PARP levels in B16F10 tumor tissues of HFD-fed mice. (Figure 2B, 2E, and 2F). However, *in vitro* cell culture studies showed that OL treatment did not reduce the viability of B16F10 melanoma cells at 2.5–10 μmol/L (Supplementary Figure 3C). Therefore, we determined the effect of 3T3-L1 conditioned media (CM) on the proliferation of B16F10 melanoma cells by using the BrdU cell proliferation assay. B16F10 cell proliferation was significantly increased by CM of adipocytes under hypoxic conditions (Supplementary Figure 3D).

IF assay results revealed that OL feeding decreased the expression of platelet endothelial cell adhesion molecule-1 (CD31), VE-cadherin (angiogenesis marker), and lymphatic vessel endothelial hyaluronan receptor (LYVE)-1 (lymphangiogenesis marker) in tumors (Figure 2G and 2H). The number of VE-cadherin-positive microvessels and LYVE-1-positive lymphatic vessels was higher in the HFD group than the CD group; and OL treatment significantly suppressed the numbers of these vessels in tumors compared with the HFD-fed group (Figure 2I and 2J). Moreover, HFD-increased mRNA expressions of CD31 and LYVE-1 were significantly suppressed in the OL-fed group (Figure 2K). However, *in vitro* treatment with OL at 2.5–10 μmol/L did not directly reduce the viability of HUVECs and LECs (Supplementary Figure 4A and 4B).

### Dietary OL decreases HFD-stimulated expression of proteins related to hypoxia, angiogenesis, and lymphangiogenesis in tumors and LNs

**Figure 3:**
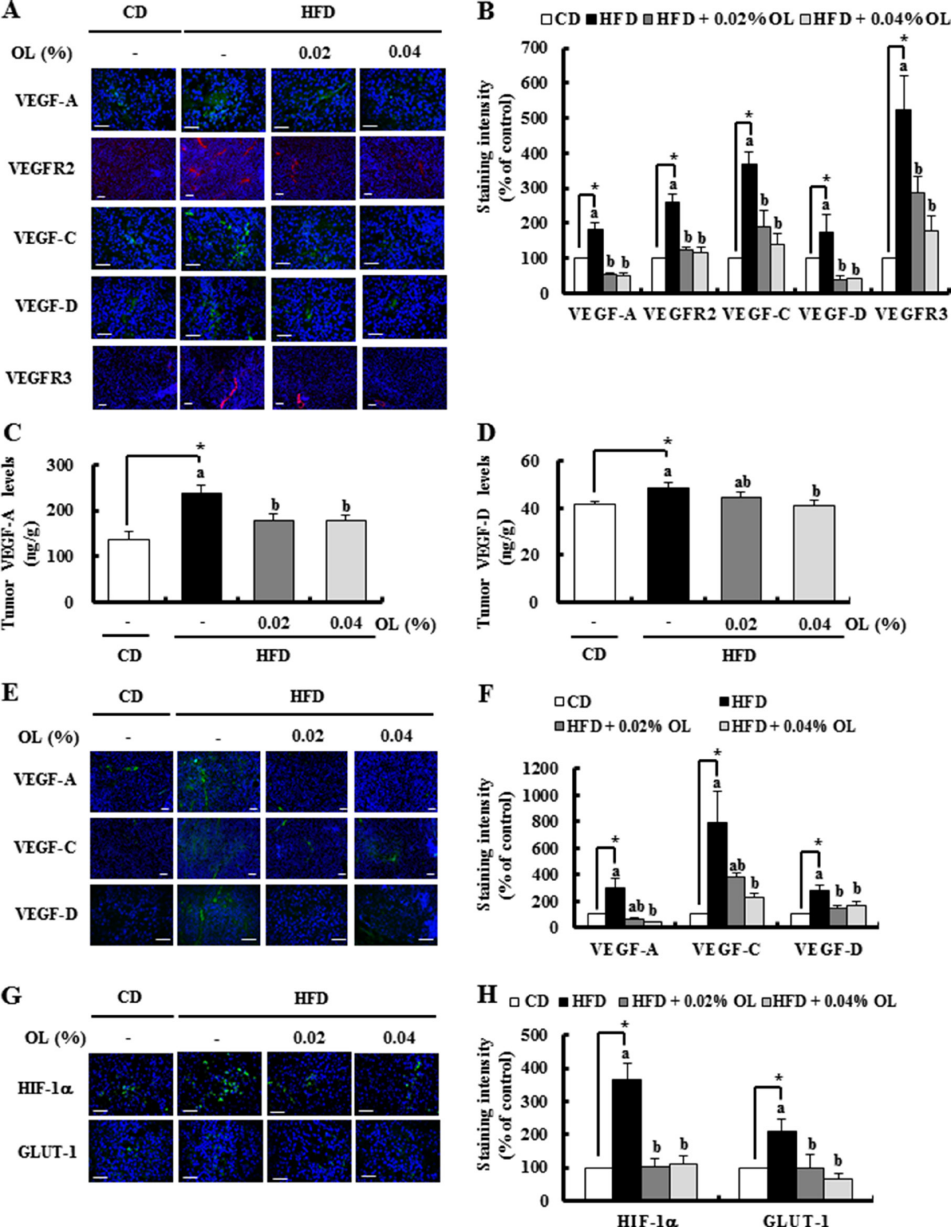
OL suppresses HFD-stimulated expression of proteins related to angiogenesis and lymphangiogenesis in the tumors and LNs (**A**, **B**, **G**, **H**) Tumor sections were stained with the indicated antibodies. (A, G) Representative immunofluorescence images. The staining intensity of (B) VEGF-A, VEGFR2, VEGF-C, VEGF-D, and VEGFR3 and (H) HIF-1α and GLUT-1 was quantified (*n* = 5–9). (**C**, **D**) The concentrations of (C) VEGF-A and (D) VEGF-D in tumor tissues were measured using ELISA (*n* = 10). (**E**, **F**) Sections of the LNs were stained with the indicated antibodies. Nuclei were counterstained with DAPI (blue). (E) Representative stained images are shown. (F) The staining intensity of VEGF-A, VEGF-C, and VEGF-D was quantified (*n* = 4). Each bar represents the mean ± SEM (*n* = 5–9). *Significantly different from the CD group, *P* < 0.05. Means without a common letter differ among the three HFD groups, *P* < 0.05.

IF staining showed that HFD feeding markedly increased the expression of VEGF-A, VEGFR2, VEGF-C, VEGF-D and VEGFR3 in the tumor tissues, which was blocked by dietary OL (Figure 3A and 3B). Enzyme-linked immunosorbent assay (ELISA) results revealed that HFD-induced increases in VEGF-A and -D levels were also decreased by OL-fed tumor tissues (Figure 3C and 3D). IF studies showed that HFD-stimulated VEGF-A, -C, and -D expression was significantly decreased in the LNs of OL-fed mice (Figure 3E and 3F). Moreover, hypoxia indices (HIF-1α and GLUT-1) were increased in the tumor tissues of HFD-fed mice, and were decreased in the tumor tissues of OL-fed mice (Figure 3G and 3H).

### Dietary OL suppresses HFD-induced accumulation M2-MΦs in the tumor tissues and adipose tissues surrounding the LNs

**Figure 4:**
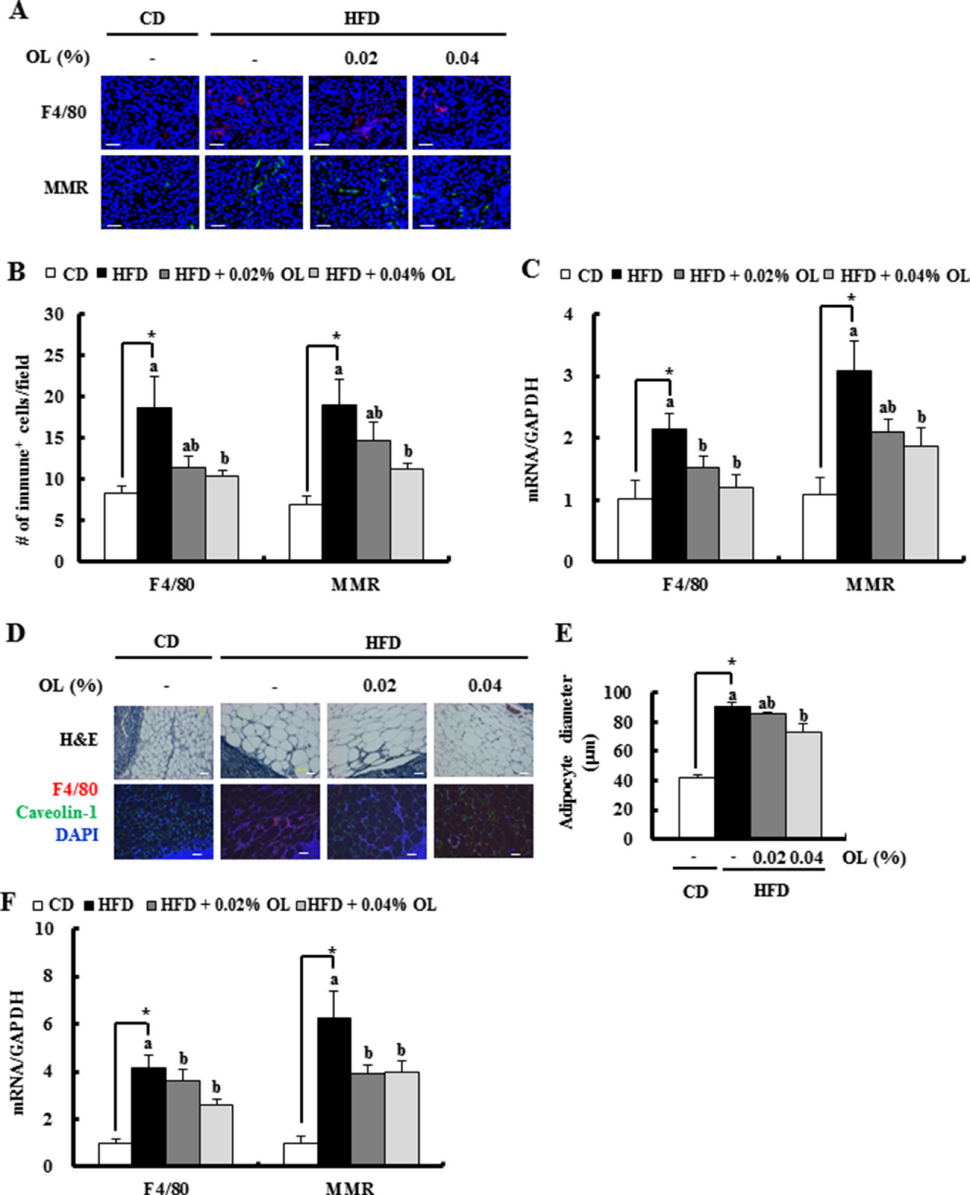
OL suppresses HFD-induced accumulation of M2-macrophages in the tumor tissues and adipose tissues surrounding the LNs (**A**, **B**) Tumor sections were stained with an antibody raised against F4/80 or MMR. (A) Representative immunofluorescence images are shown. (B) F4/80 and MMR-positive cells were quantified (*n* = 5). (**C**) Total RNA was isolated from tumor tissues, and F4/80 and MMR gene expression levels were determined using real-time RT-PCR (*n* = 9). (**D**, **E**) Sections of adipose tissues surrounding the LNs were stained with hematoxylin and eosin. Mature macrophages were identified using an F4/80 antibody (red) and adipocyte membranes a caveolin-1 antibody (green). Nuclei were counterstained with DAPI (blue). (D) Representative stained images are shown. (E) Mean diameters of adipocytes surrounding the LN were estimated (*n* = 4). (**F**) F4/80 and MMR gene expression levels in fat tissues surrounding the LN, as determined using real-time RT-PCR (*n* = 6). *Significantly different from the CD group, *P* < 0.05. Means without a common letter differ among the three HFD groups, *P* < 0.05.

IF staining revealed that HFD-induced increases in F4/80-positive mature MΦs and murine mannose receptor (MMR)-positive M2-MF infiltration in tumor tissues were decreased by OL supplementation (Figure 4A and 4B). Real-time RT-PCR assays showed that the HFD-induced increased F4/80 and MMR mRNA levels were also decreased in OL-fed tumor tissues (Figure 4C). Moreover, HFD consumption increased the size of adipocytes and the number of F4/80- positive macrophages in fat tissues surrounding the LNs, and these effects were blocked by OL feeding (Figure 4D and 4E). F4/80 and MMR mRNA levels were increased in fat tissues surrounding the LNs in HFD-fed mice. The HFD feeding-induced upregulation of F4/80 and MMR mRNA levels was reduced by OL supplementation (Figure 4F).

### Co-culture of B16F10s, M2-MΦs and 3T3-L1 adipocytes stimulates the production of VEGF-A and -D which promote angiogenesis and lymphangiogenesis under hypoxic conditions

**Figure 5:**
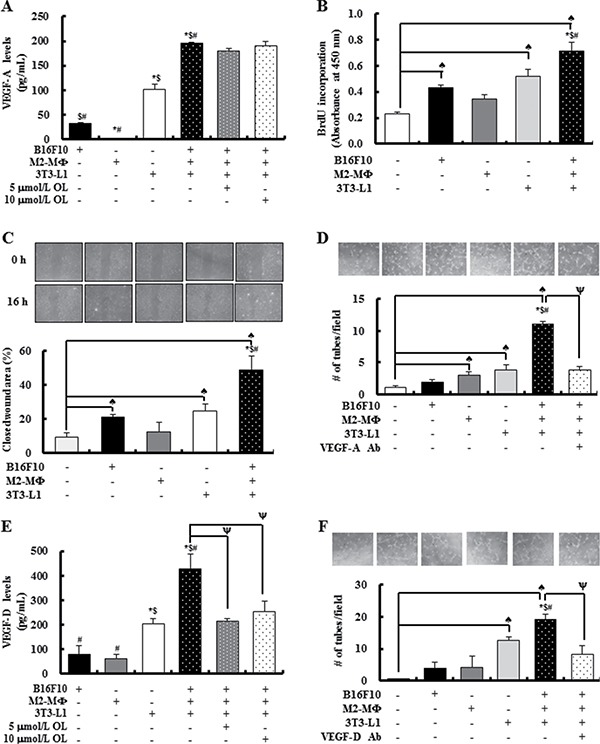
The cross-talk between tumor cells, M2-cells and adipocytes results in release of substantial amounts of VEGF-A and VEGF-D under hypoxic conditions stimulating angiogenesis and lymphangiogenesis, respectively B16F10s, M2-MΦs, and 3T3-L1 adipocytes were cultured separately or co-cultured under hypoxic conditions (1% O_2_, 5% CO_2_, and 94% N_2_) with or without OL treatment (0 - 10 μmol/L). (**A**) The concentrations of VEGF-A in conditioned media (CM) were estimated by using ELISA. Each bar represents the mean ± SEM (*n* = 4). (**B**) Cell proliferation was measured by using BrdU incorporation assay. HUVECs were treated for 6 h with CM collected under hypoxic conditions (hypoxic-CM). BrdU was then added, and the incubation was continued for a further 3 h to analyze BrdU incorporation into DNA (*n* = 3). (**C**) For wound-healing assay, HUVECs were plated and grown to 100% confluence; subsequently, an injury line was created using a yellow pipette tip and the cells were treated with hypoxic-CM for 16 h. (Upper panel) Wound closure was visualized under phase-contrast microscopy. (Lower panel) Quantification of wound closure (*n* = 3). (**D**) For tube formation assay, HUVECs were plated in Matrigel-coated plates. After 24 h, HUVECs were treated with hypoxic-CM in the absence or presence of an anti-VEGF-A antibody. (Upper panel) Tube formation was visualized under phase-contrast microscopy. (Lower panel) Quantification of HUVEC tube formation (*n* = 3). (**E**) VEGF-D concentrations in CM were estimated using ELISA (*n* = 4). (**F**) For tube formation assays, LECs were treated with hypoxic-CM in the absence or presence of an anti-VEGF-D antibody. (Upper panel) Tube formation was visualized under phase-contrast microscopy. (Lower panel) Quantification of LEC tube formation. Each bar represents the mean ± SEM (*n* = 3). ^♠^Significantly different from the DMEM group; *significantly different from the B16F10 group; ^$^significantly different from the M2-MΦ group; ^#^significantly different from the 3T3-L1 group; and ^ψ^significantly different from the B16F10/M2-MF/3T3-L1 co-culture group, *P* < 0.05.

The results of the study using tumor-bearing mice indicated that dietary OL suppressed HFD-stimulated angiogenesis and lymphangiogenesis, accompanied by a decrease in VEGF expression (Figure 3). We next examined the mechanisms by which dietary OL suppresses tumor angiogenesis and lymphangiogenesis using *in vitro* cell culture studies. To determine whether the changes in adipocytes and M2-MΦs in the tumor microenvironment are important for angiogenesis and lymphangiogenesis, we designed a direct co-culture system of B16F10, M2-MΦs, and differentiated 3T3-L1 cells at a 2:1:1 ratio under hypoxic conditions. B16F10s, M2-MΦs, and 3T3-L1 adipocytes produced VEGF-A, and 3T3-L1s produced the highest levels (Figure 5A). VEGF-A production was markedly increased in the direct co-culture CM of the three cells as compared to that of the three cell types individually. However, OL treatment did not inhibit the co-culture-induced VEGF-A production. A BrdU incorporation assay revealed that HUVEC cell proliferation was significantly increased by B16F10 and 3T3-L1 CM, and further increased by the co-culture CM (Figure 5B). To examine the effects of the co-culture CM on angiogenesis, wound migration and tube formation assays were performed using HUVEC cells. Co-culture CM of B16F10s, M2-MΦs, and differentiated 3T3-L1 cells significantly induced migration and tube formation by HUVECs (Figure 5C and 5D). Addition of a VEGF-A neutralizing antibody (200 pg/mL) to the co-culture CM markedly inhibited tube formation of HUVECs (Figure 5D).

VEGF-D, an important growth factor for lymphangiogenesis, showed increased levels in direct co-culture CM, as compared to that of the three cell types individually; and OL treatment directly inhibited these increases in VEGF-D production (Figure 5E). In addition, tube formation by LEC cells was significantly increased by the co-culture CM, which was strongly diminished by addition of VEGF-D neutralizing antibody (400 pg/mL) (Figure 5F). The direct effect of OL on angiogenesis and lymphangiogenesis was investigated *in vitro* using HUVECs and LECs. OL dose-dependently inhibited tube formation by HUVECs and LECs (Supplementary Figure 4C and 4D). These results indicated that OL indirectly and directly inhibits angiogenesis and lymphangiogenesis in obesity-induced tumor progression.

## DISCUSSION

Epidemiological data suggest that obesity is a risk factor for cancer [3, 33–35] and increased body weights is positively associated with increased death rates for all cancers combined [4]. Moreover, our previous study demonstrated that solid tumor growth and metastasis were markedly accelerated in mice with HFD-induced obesity [5]. As the prevalence of overweight and obesity has increased rapidly worldwide, development of effective agents that suppress obesity-stimulated cancer progression is necessary. The results of the present study indicated that dietary OL inhibits tumor growth and LN metastasis in HFD-fed mice bearing B16F10 melanoma cells, which is associated with the inhibition of angiogenesis and lymphangiogenesis. Our results indicated that these effects are due to (i) inhibition of adipocyte differentiation and infiltration of M2-MΦs; (ii) decreases in HIF-1α levels; (iii) reductions in the levels of VEGF-A and -D; and (iv) direct inhibition of endothelial and lymphatic endothelial cells. Thus, OL has potential as a candidate preventive agent for use against obesity-stimulated cancer progression.

### OL inhibition of adipogenesis

Several previous reports have shown that OL inhibits adipogenesis. The results of Kuem *et al*. [36] and the present study (Supplementary Figures 2B, 2C, and 3A) revealed that OL treatment inhibits differentiation of 3T3-L1 adipocytes *in vitro* and reduces fat mass *in vivo*. Additionally, Kuem *et* *al*. showed that OL reversed HFD-induced elevations of gene expression involved in WNT10b-mediated signaling in adipose tissue of mice [36]. Moreover, in a time-course analysis of the effect of OL, Drira *et al*. reported that OL exerts its effects during the early stages of 3T3-L1 differentiation. In their study, OL reduced the expression of peroxisome proliferator-activated receptor g and CCAAT-enhancer-binding protein a, the major transcription factors in adipocyte differentiation [37]. Furthermore, dietary intake of olive leaf extract has been shown to improve insulin sensitivity in middle-aged overweight men [38]. Consistent with these results, the current findings revealed that dietary OL reduced fat accumulation and HOMA-IR in mice fed a HFD (Supplementary Figure 2B–2F). Taken together, these results indicate that OL has direct inhibitory effects on adipogenesis, leading to inhibition of tumor progression, insulin resistance, etc.

### OL inhibition of HFD-induced tumor angiogenesis and lymphangiogenesis

In tumor-bearing mice, OL inhibited HFD-induced tumor angiogenesis and lymphangiogenesis (Figure 2G–2K). *In vitro* results revealed that OL directly inhibited tube formation by HUVECs and LECs (Supplementary Figure 4C and 4D). OL treatment (10 μmol/L) of HUVECs has been reported to inhibit PMA-induced tube formation and migration by decreasing the activity of nuclear factor kB (NF-kB) and its downstream targets, MMP-9 and COX-2 [39], indicative of its direct inhibitory effect on endothelial cells.

Rapidly growing tumors exceed their vascular supply and become hypoxic. The hypoxia response is largely mediated by HIFs. HIF-1α accumulation leads to upregulation of genes that are involved in many aspects of cancer progression, including metabolic adaptation, apoptosis resistance, angiogenesis, lymphangiogenesis and metastasis (reviewed in [40, 41]). The present *in vivo* results revealed that OL decreased HFD-induced HIF-1α and GLUT-1 levels in tumor tissues (Figure 3G and 3H). A previous study showed that OL downregulates HIF-1α in HT-29 colon cancer cells [42] and NF-κB directly interacts with HIF-1α to regulate HIF-1α mRNA and protein levels [43, 44]. These results support the hypothesis that dietary OL downregulates HFD-induced HIF-1α, which may play a role in the regulation of HFD-induced angiogenesis, including downregulation of VEGFs.

VEGF-A is an important mediator of tumor angiogenesis. This effect is mediated via VEGFR2, resulting in promotion of angiogenesis, vascular permeability, cell migration, and gene expression (reviewed in [45]). VEGF-C and VEGF-D regulate lymphangiogenesis via VEGFR3 [9]. Overexpression of VEGF-C/D in murine orthotopic models leads to an increased number of peritumoral lymphatic vessels and enhanced metastasis to regional LNs (reviewed in [46]). Additionally, blocking of the VEGF or the VEGF receptor inhibits angiogenesis and suppresses the growth of many types of tumors in experimental models [47, 48]. In the present study, we first demonstrated that the HFD-induced expression of VEGF-A, -C and -D, and VEGFR-2 and -3 was suppressed by dietary OL supplementation (Figure 3A and 3B). These results revealed that OL suppresses HFD-induced stimulation of the VEGF-A/VEGFR2 and VEGF-C/−D/VEGFR3 axis, and thereby inhibits angiogenesis and lymphangiogenesis in melanoma-bearing HFD-fed mice.

As VEGFs/VEGFRs were significantly induced in tumor tissues in HFD-fed mice (Figure 3), we examined whether cells in the tumor microenvironment secrete VEGF-A and VEGF-D using *In vitro experiments*. Differentiated 3T3-L1 cells secreted significant quantities of VEGF-A and VEGF-D. Co-culture results showed that the crosstalk between B16F10, M2-MΦs, and differentiated 3T3-L1 cells, especially under hypoxic conditions, stimulated the production of VEGF-A and VEGF-D and enhanced proliferation, migration, and tube formation by endothelial cells. An antibody against VEGF-A or VEGF-D abrogated the co-culture CM-induced stimulation of tube formation by HUVECs and LECs (Figure 5). These results indicate that VEGF-A and VEGF-D produced by these three cell types stimulates tumor angiogenesis and lymphangiogenesis. Our previous work showed that mature adipocytes increased the expression of VEGF-D in M2-MΦs. The expression of VEGFR3 in LECs was increased by adipocytes [5]. These results also support the importance of adipocytes as stimulators of tumor angiogenesis and lymphangiogenesis. Co-culture-induced VEGF-A production was not abrogated by OL treatment (Figure 5A). Thus, OL suppression of adipocyte accumulation could possibly result in decreased VEGF-A levels in tumor tissues. In contrast to VEGF-A, OL directly inhibited co-culture-induced VEGF-D production (Figure 5E), indicating that it inhibits VEGF production by both decreasing adipocyte accumulation and exerting direct inhibitory effects on these cells. Collectively, these findings indicate that the inhibitory effects of OL on tumor angiogenesis and lymphangiogenesis in obese mice are, at least in part, due to the inhibition of adipose tissue accumulation leading to a reduction in VEGF/VEGFR production.

Obesity increases the number of stromal inflammatory cells, including F4/80-positive MΦs, leading to the stimulation of ovarian cancer cell growth *in vivo* [49]. We have reported previously that HFD stimulates infiltration of M2-MΦs in B16F10 tumor tissues. Adipocytes induce CCL-2 and M-CSF expression in B16F10s, which increases the number of M2-MΦs in the tumor. M2-MΦs express VEGF-D and promote VEGF-A expression in B16F10 cells. Additionally, the crosstalk between B16F10s and M2-MΦs further increases the levels of several cytokines and angiogenic and lymphangiogenic factors [5]. Therefore, blockage of tumor infiltration by M2-MΦs can suppress the induction of tumor angiogenesis in obese cancer patients. The present study revealed that OL supplementation significantly inhibited infiltration of F4/80-positive and MMR-positive M2-MΦs in tumor tissues and adipose tissues surrounding the LNs in HFD-fed mice (Figure 4). *In vitro* studies revealed that M2-MΦs secreted VEGF-A and VEGF-D, and M2-MF CM stimulated tube formation by HUVECs. Co-culture results indicated that the crosstalk between tumor cells, M2-MΦs, and mature adipocytes under hypoxic conditions stimulates the production of VEGF-A and VEGF-D and enhances the proliferation, migration and tube formation of endothelial cells (Figure 5). These results suggested that the inhibition of M2-MF infiltration into tumor tissues by dietary OL contributes to the reduced angiogenesis in these animals.

### OL inhibition of HFD-induced solid tumor growth

Hamdi *et al*. reported that OL treatment (1% in drinking water) for 9–12 days inhibited tumor growth and induced tumor regression in Swiss albino mice that spontaneously develop soft tissue sarcomas [32]. In the present study, OL decreased the HFD-induced expression of CDK4/cyclin D1, inhibited HFD-induced reduction of apoptotic cell numbers, and inhibited the HFD-induced decreases in the levels of cleaved PARP in B16F10 melanoma tumor tissues (Figure 2A–2F). Previous *in vitro* studies have demonstrated that OL inhibits the proliferation and migration of several tumor cell lines *in vitro* at 0.005–0.025% (92.5–462.5 μmol/L) [32]. OL (370 μmol/L) reduced breast cancer cell growth [50] and the expression levels of MMP-9 and MMP-2, and induced apoptotic cell death of breast cancer cells [51]. It has also been reported that OL induces G1 cell cycle arrest in MCF7 breast cancer cells [50] and G2/M phase arrest and apoptotic cell death in HeLa cells [52]. Seçme *et al*. reported that OL treatment changes the expression of cell cycle- and apoptosis-related genes in SH-SY5Y neuroblastoma cells [53]. These *in vitro* results obtained using higher concentrations of OL (40–460 μmol/L) indicate that OL directly regulates cell cycle progression and apoptosis of tumor cells. However, we noted that OL did not directly inhibit B16F10 melanoma cell proliferation at lower concentrations (2.5–10 μmol/L) *in vitro* (Supplementary Figure 3C), whereas dietary OL reduced tumor growth in melanoma cell-bearing mice *in vivo* (Figure 1). We did not determine the bioavailability of OL in the present study. Del Boccio *et al*. reported that plasma concentrations of OL peaked at 200 ng/mL (0.37 μmol/L) 2 h after a single oral dose of OL (100 mg/kg body weight) in rats [54]. As we continuously supplied OL containing diet (20 or 40 mg/kg/day/mouse assuming that the mouse consumed 3 g of diet/day), it is expected that OL concentrations in the blood of our mice were maintained at nanomolar concentrations. Therefore, it is reasonable to assume that OL inhibits melanoma cell proliferation via indirect mechanisms in HFD-fed mice.

Decreased angiogenesis can explain the decreased cancer cell proliferation because new blood vessels provide nutrients and oxygen to rapidly growing cancer cells. OL reduces the number of lipid vacuoles in tumor tissues and the size of adipocytes in adipose tissues surrounding the LNs. Recently, Cohelo *et al*. [21] reported that 3T3-L1 and adipose tissue CM increases B16F10 cell survival by enhancing their proliferation and decreasing apoptosis. We also noted that BrdU incorporation in B16F10 cells was significantly increased by hypoxic 3T3-L1 CM (Supplementary Figure 3D). Thus, the decrease in adipocytes may be another reason for the decreased tumor cell proliferation. Additionally, OL decreased the numbers of M2-MΦs in tumor tissues and adipose tissues surrounding the LN (Figure 4). We have reported previously that M2-MΦs stimulates the proliferation of 4T1 mammary carcinoma cells [55]. Growth-stimulating effects of M2-MΦs on several other cancer cells have been also reported [56, 57]. Taken together, these results suggest that dietary OL used in the present study induces cell cycle arrest and apoptosis in tumor tissues of HFD-fed mice via indirect mechanisms, including inhibition of adipocyte differentiation, infiltration of M2-MΦs, and angiogenesis.

It is well known that cholesterol-laden macrophages (foam cells) are found in atherosclerotic lesions. Similarly, Prieur *et al*. have shown that M1-polarized adipose tissue macrophages are lipid-loaded and resemble proatherosclerotic foam cells. These foam cells contain many small lipid vesicles unlike adipocytes which contain a large lipid droplet. In the present study, the morphology and functions of lipid-laden M1-macrophages were not determined. Future studies are needed to examine the role(s) of foam cells in tumor metastasis [58].

In the present study, we demonstrated that dietary OL suppresses solid tumor growth and LN metastasis of B16F10 melanoma cells in C57BL/6 mice. This study also provided experimental evidence that dietary OL supplementation inhibits tumor angiogenesis and lymphangiogenesis. *In vitro* results revealed that OL directly inhibited adipocyte differentiation. Dietary OL inhibited HFD-induced accumulation of adipocytes and M2-MΦs, expression of VEGF-A, -D, and HIF-1α in tumor tissues, thereby suppressing tumor angiogenesis and lymphangiogenesis in melanoma-bearing obese mice. These results suggested that the inhibition of adipogenesis by OL contributes to its beneficial effects against melanoma tumor growth and metastasis in obesity. However, the detailed mechanisms by which OL reduces the levels of VEGFs and HIF-1α, and the crosstalk between cancer cells, M2-MΦs and adipocytes remain to be elucidated.

## MATERIALS AND METHODS

### Reagents

Oleuropein (purity = 98.0%) was purchased from Extrasynthese (Genay, France). Antibodies against caveolin-1, F4/80, GLUT-1, Ki67, LYVE-1, VE-cadherin, VEGF-D and VEGFR3 were obtained from Abcam (Cambridge, MA, UK). HIF-1α, VEGF-A, VEGF-C, MMR, CDK4, cyclin D1 and CD31 antibodies were purchased from Santa Cruz Biotechnology (Santa Cruz, CA, USA). Cleaved PARP and VEGFR2 antibodies were obtained from Cell Signaling (Beverly, MA, USA). Matrigel was purchased from Corning (MA, USA).

### Cell culture

B16F10 and 3T3-L1 cells were purchased from American Type Culture Collection (Manassas, MA, USA). B16F10 cells were maintained in Dulbecco's Modified Eagle's Medium (DMEM) supplemented with 100 ml/L fetal bovine serum (FBS), 100,000 U/L of penicillin and 100 mg/L of streptomycin. 3T3-L1 cells were maintained in DMEM supplemented with 100 ml/L calf serum, 100,000 U/L of penicillin and 100 mg/L of streptomycin. HUVECs and LECs were purchased from Lonza (Walkersville, MD, USA). HUVECs and LECs were maintained in Endothelial Cell Basal Medium (EBM)-2 supplemented with EGM-2 Single Quots (Lonza, Walkersville, MD, USA). For all experiments, cells were used within 10 passages after arrival.

### Animal experiments

The experimental design is shown in Supplementary Figure 1. This animal study was approved by the Institutional Animal Care and Use Committee of Hallym University (Hallym 2015–40) and conducted in accordance with its guidelines.

Three-week old, male C57BL/6N mice were obtained from Orient Bio (Gapyung, Korea). All mice were fed an AIN-76A diet (Research Diets, Inc., New Brunswick, NJ, USA) and water *ad libitum* for 1 week as an adaptation period. The mice were then randomly divided into four groups (*n* = 20/group) and fed the following diet during the entire experimental period: CD (10 kcal% of fat), HFD (60 kcal% of fat), HFD + 0.02% of OL, or HFD + 0.04% of OL. The nutritional compositions of the CD and HFD (Research Diets, Inc., New Brunswick, NJ, USA) are reported in Supplementary Table 1. Fourteen weeks after diet feeding, fat and lean body mass were measured using a Lunar PIXImus densitometer (GE Lunar, WI, USA). Sixteen weeks after diet feeding, B16F10-luc cells [5 × 10^4^ cells in 0.1 mL Matrigel/PBS (Corning, MA, USA)] were subcutaneously injected into the right rear flanks of the mice. Three weeks after melanoma cell injection, the solid tumors were surgically resected and weighed. Seventeen days later, all animals were euthanized, the draining LNs were resected and weighed. Metastasized melanomas in the LNs were also dissected and weighed. The blood was collected and plasma was isolated by centrifugation. All tissue samples were stored at −80°C.

The growth of solid tumors and LN metastasis were assessed by *in vivo* BLI. For BLI, mice were intraperitoneally injected with 150 mg/kg bodyweight of D-luciferin substrate (GoldBio, St. Louis, MO, USA) suspended in PBS and imaged within 30 min using an *in vivo* imaging system-200 (Caliper Life Sciences, Hopkinton, MA).

### Preparation of differentiated 3T3-L1 cells and Oil Red O staining

For 3T3-L1 cell differentiation, cells were plated at 6 × 10^4^ cells/well in 24-well plates. Two days after full confluence (day 0), cells were incubated in DMEM supplemented with 100 ml/L FBS, 100,000 U/L of penicillin and 100 mg/L of streptomycin containing 1 μmol/L dexamethasone, 0.5 mM isobutylmethylxanthine, and 10 μg/mL insulin (DMI) for 48 h, and for 48 h in DMEM containing 10% FBS and 10 μg/mL insulin. They were then cultured in DMEM containing FBS and the antibiotics. The cells were exposed to OL (0 −10 μmol/L) between days 0 and 8. On day 8, lipid accumulation was detected by Oil Red O staining as described previously [59]. Briefly, samples were fixed in 4% paraformaldehyde for 1 h and stained with Oil Red O for 2 h. Images were then captured under a microscope. The dye was extracted with isopropanol, and the absorbance (490 nm) was measured by a microplate reader (Bio-Rad, CA, USA).

### Measurement of glucose and insulin levels

Fasting glucose and insulin were determined in overnight-fasted mice. Blood samples were obtained through orbital eye bleeding for analyses of plasma insulin and glucose levels. The levels of plasma glucose and insulin were measured using a glucose colorimetric assay kit (Cayman Chemicals, Ann Arbor, MI, USA) and an insulin ELISA kit (Millipore, Billerica, MA, USA) following the manufacturer's instructions, respectively. Insulin resistance (IR) was assessed using the homeostasis model: The HOMA-IR = fasting glucose level (mmol/L) × fasting insulin level (mU/L) ÷ 22.5 [60].

### MTT assay

Cell viability was measured using mitochondrial dehydrogenase 3-(4,5-dimethylthiazol-2-yl)-2.5-diphenyl tetrazolium bromide (MTT, Sigma, MO, USA). MTT assay was performed as described previously [61]. Briefly, the cells (3T3-L1, B16F10, HUVEC, LEC) were plated in 24-well plates (HUVECs, 5 × 10^4^ cells; LECs, 4 × 10^4^ cells, B16F10 cells, 2.5 × 10^4^ cells; 3T3-L1 cells, 6 × 10^4^ cells) with or without OL (2.5–10 μmol/L) and incubated for the indicated times. MTT solution was then added and the cells were incubated for a further 3 h. The optical density was read at 570 nm using a microplate reader (Bio-Rad, CA, USA).

### IF staining and TUNEL assay

For IF processing, 8 μm frozen sections were fixed for 10 min in methanol and rehydrated in PBS. The slides were incubated with protein blocking solution (5% bovine serum albumin in TBST) for 1 h at room temperature, then overnight at 4°C with a primary antibody. After washing with PBS, a fluorochrome-conjugated secondary antibody (Alexa-488, 594 or 633) was used and nuclei were counterstained with 4′6-diamidino-2-phenylindole (DAPI; Sigma, MO, USA). All sections were observed under a microscope and analyzed with the AxioVision digital image processing software (Carl Zeiss, Jena, Germany). Microvessel density was evaluated by IF detection of VE-cadherin-positive vessels and lymphatic vessel density was determined by IF staining of LYVE-1-positive vessels. Six fields of each tissue section were analyzed for microvessel density at ×200 magnification. To assess apoptosis, TUNEL staining (TUNEL, Promega, Madison, WI, USA) was performed according to the manufacturer's instructions.

### ELISAs

Tumor lysates were prepared [62] and the levels of VEGF-A and VEGF-D in tumor tissues were estimated using the relevant ELISA kits (R&D Systems) according to the manufacturer's instructions.

### Real-time RT-PCR analysis

Total RNA was isolated from tissues using TRIzol reagent (Roche, Indianapolis, IN, USA). After extraction of total RNA, cDNA was synthesized from 1 μg of total RNA with a Maxime RT PreMix kit (iNtRON Biotechnology, Gyeonggi, Korea) according to the manufacturer's instructions. The PCR reaction was conducted using PCR premix (Bioneer Co., Daejeon, Korea) with the appropriate sense/antisense primers specific for the genes indicated in Supplementary Table 2. Real-time RT-PCR was performed using a LightCycler 480 SYBR green I Master instrument (Roche, Mannheim, Germany). The resulting data were analyzed using the LighCycler^®^ 480 II software (Roche, Mannheim, Germany).

### Co-culture of B16F10, M2-MΦs and 3T3-L1 under hypoxic conditions

For M2-MF preparation, Raw264.7 cells were treated with 10 ng/mL recombinant mouse interleukin-4 (R&D Systems, Minneapolis, MN, USA) in DMEM supplemented with 100 ml/L FBS, 100,000 U/L of penicillin and 100 mg/L of streptomycin for 24 h. For co-culture experiments, 3T3-L1 cells (6 × 10^4^/well) were plated and differentiated in 24-well plates. Then, B16F10 (1.2 × 10^5^ /well) cells and/or M2-MΦs (6 × 10^4^ /well) were added to differentiated 3T3-L1 cells (i.e., a cell ratio of 2:1:1). After 24 h, the cells were serum-starved for 4 h in DMEM. To expose the cells to hypoxic conditions, they were incubated in a modular incubator chamber (Sanyo Electric Co., Moriguchi, Osaka, Japan), infused with a mixture of 1% O_2_, 5% CO_2_, and 94% N_2_. After 8 h of exposure to hypoxia, CM were collected for further analyses.

### BrdU assay

Cell proliferation was assessed using a BrdU Cell Proliferation Assay kit (BioVision, Milpitas, CA, USA) according to the manufacturer's instructions. In brief, HUVECs were plated in 96-well plates at a density of 6 × 10^4^ /well in EBM-2 supplemented with EGM-2 Single Quots, and serum-starved in EBM-2 for 2 h. After serum-starvation, the cells were treated with the co-culture CM for 6 h. BrdU was then added and after 3 h incubation, BrdU incorporation into DNA was measured using a microplate reader (Bio-Rad, CA, USA).

### Wound-healing migration assay and tube formation assay

HUVECs (4.8 × 10^5^ cells/well) were plated in six-well plates in EBM-2 supplemented with EGM-2 Single Quots. Upon reaching confluence, the cell layer was scratched with a yellow pipette tip. The cells were incubated with the co-culture CM. After 12 h of incubation, the number of cells that had migrated into the gap was determined, and the percent closure of each wound was calculated. For tube formation assays, HUVECs (5 × 10^4^ cells/well) and LECs (4 × 10^4^ cells/well) were seeded in EBM-2 (Lonza, MD, USA) into 24-well plates pre-coated with 0.3 mL of Matrigel. OL (0–10 μmol/L) or co-culture CM was added to the wells, the plates were incubated for 4 h, and tube formation was determined. For quantification of angiogenesis, the total length of formed tubes was measured using the Motic Images Advanced 3.2 system (Motic, Richmond, BC, Canada).

### Statistical analysis

Statistical analysis was performed using the Statistical Analysis System for Windows, version 9.4 (SAS institute, Cary, NC). All data were expressed as means ± SEM. The statistical significance of differences was evaluated by applying Student's *t-test* for comparisons between two groups (such as the CD and HFD groups) or one-way ANOVA followed by Duncan's multiple-range test for comparisons among three groups (such as the HFD, HFD + 0.02% OL, and HFD + 0.04% OL groups). Differences were considered significant at values of *P* < 0.05.

## SUPPLEMENTARY MATERIALS FIGURES AND TABLES


