# Supplementary material for: Dietary oleuropein inhibits tumor angiogenesis and lymphangiogenesis in the B16F10 melanoma allograft model: a mechanism for the suppression of high-fat diet-induced solid tumor growth and lymph node metastasis
Source: Oncotarget. 2017 Mar 31;8(19):32027–42. doi: 10.18632/oncotarget.16757 (PMC5458266; doi:10.18632/oncotarget.16757)
Supplement: Supplementary file 1 [file oncotarget-08-32027-s001.pdf]

Dietary oleuropein inhibits tumor angiogenesis and lymphangiogenesis in the B16F10 melanoma allograft model: a mechanism for the suppression of high-fat diet-induced solid tumor growth and lymph node metastasis

Supplementary Materials

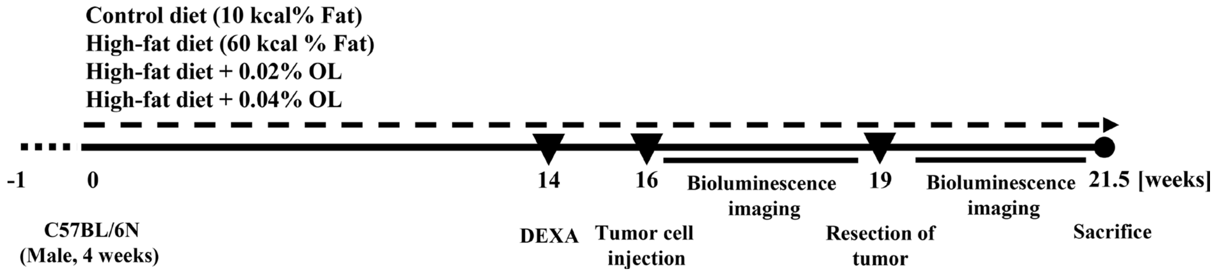

Supplementary Figure 1: Experimental design. Four-week-old, male C57BL/6N mice were fed a diet [control diet (10 kcal% fat), high-fat diet (HFD, 60 kcal% fat), HFD + 0.02% oleuropein (OL), or HFD + 0.04% OL] for the entire period. Fat and lean body mass (DEXA) were measured at 14 weeks after the initiation of feeding. Sixteen weeks after diet feeding, B16F10 melanoma cells (B16F10-luc cells, 5×10^4 cells/mouse) were subcutaneously injected into the animals' left flank. Bioluminescence imaging (BLI) was conducted at 5, 10, and 17 days after B16F10-luc cell injection. The primary tumor was resected 3 weeks after cell injection, and BLI was conducted at 10 and 15 days after tumor resection. All animals were euthanized 17 days (2.5 weeks) after the resection. The draining lymph nodes were removed and weighed.

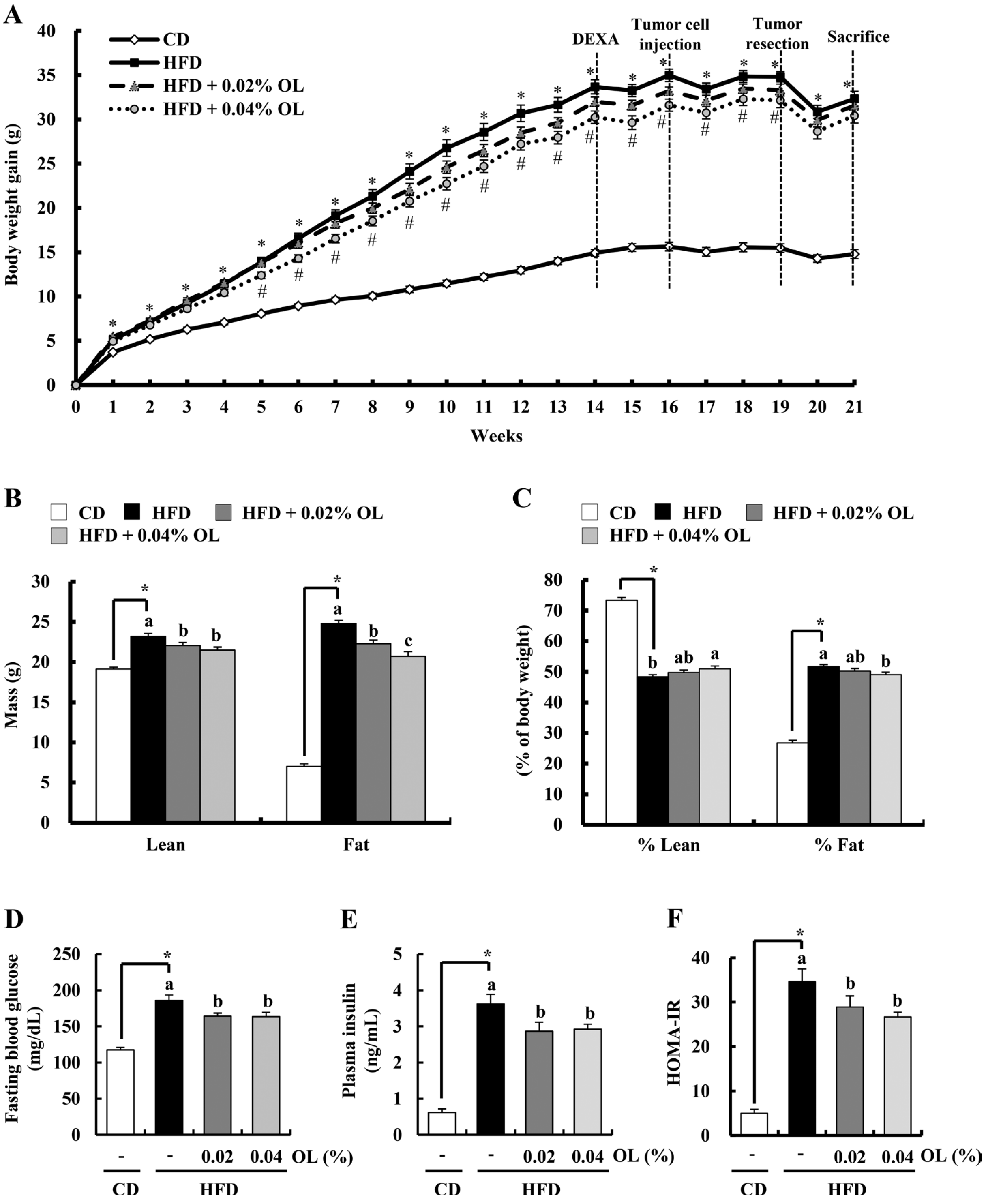

Supplementary Figure 2: OL decreases the fat mass of C57BL/6N mice. (**A**) Body weights of mice were measured weekly. Each point represents the mean ± SEM (n = 20). #Significantly different from the CD group, P < 0.05. #Significantly different from the HFD-fed group, P < 0.05 (**B**, **C**) Fourteen weeks after feeding, fat and lean body mass were measured using a Lunar PIXImus densitometer (mean ± SEM, n = 15). (**D**–**F**) At 21.5 weeks after feeding, fasting blood samples were obtained by orbital bleeding after overnight fasting for analysis of plasma (D) glucose and (E) insulin levels. (F) HOMA-IR = fasting glucose level (mmol/L) × fasting insulin level (mU/L) ÷ 22.5. Each bar represents the mean ± SEM (n = 10). (B–F) *Significantly different from the CD group, P < 0.05. Means without a common letter differ among the three HFD groups, P < 0.05

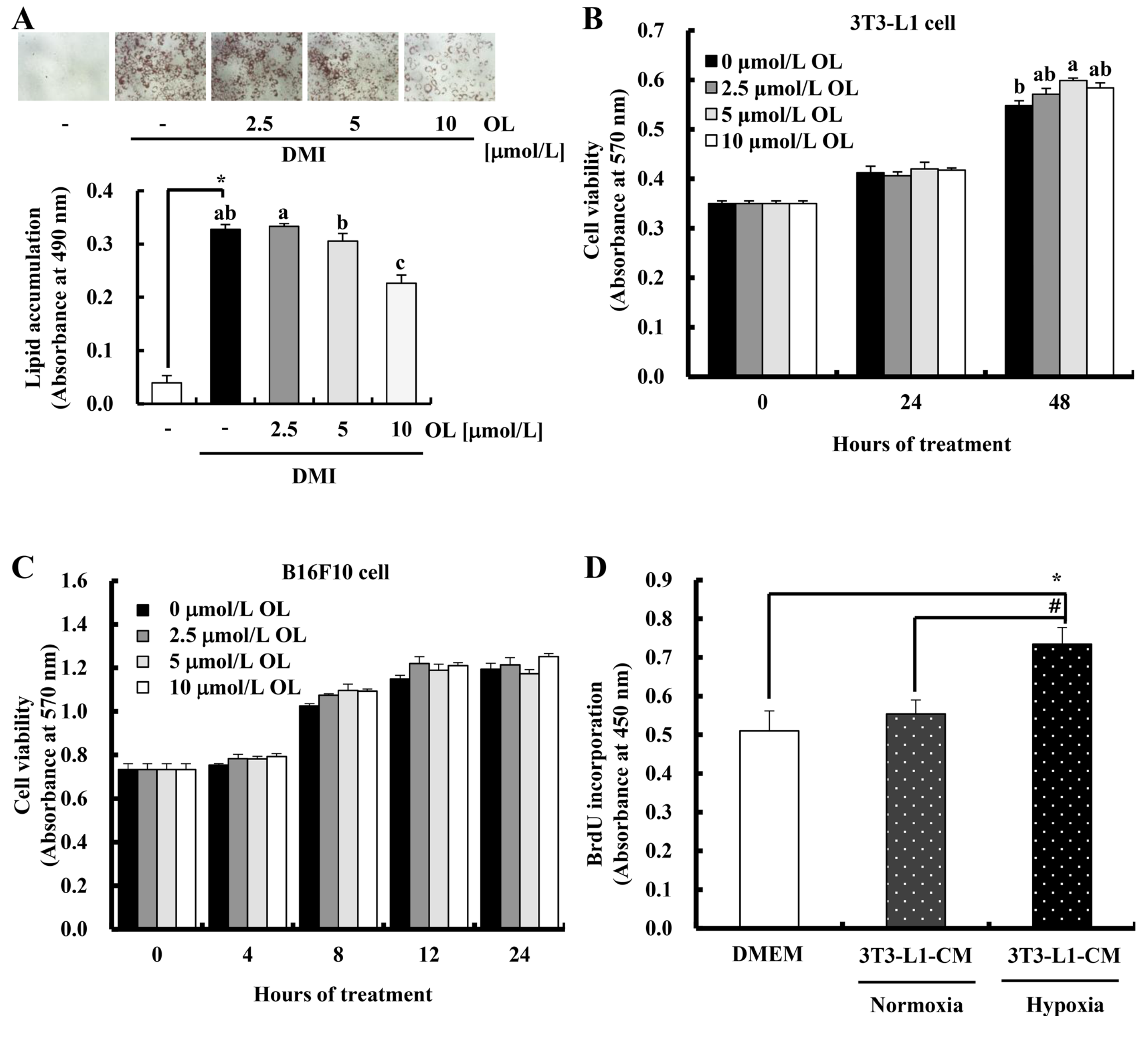

Supplementary Figure 3: OL suppresses the accumulation of lipid droplets in 3T3-L1 cells. (**A**) Confluent 3T3-L1 preadipocytes were differentiated with 1 μmol/L dexamethasone, 0.1 mmol/L 3-isobutyl-1-methylxanthine, and 10 mg/L insulin (DMI). OL (0–10 μmol/L) was present throughout the differentiation period (8 days). (Upper panel) Cells were stained with Oil Red O and visualized under phase-contrast microscopy. (Lower panel) Oil Red O-stained lipid droplets were extracted with isopropyl alcohol, and the Oil Red O staining intensity was colorimetrically quantified (mean ± SEM, *n* = 4). *Significantly different between the control (undifferentiated 3T3-L1 cells) and DMI + 0 μmol/L OL-treated cells, $P < 0.05$. Means without a common letter differ among the four DMI groups, $P < 0.05$. (**B**) 3T3-L1 cells were plated at a density of 6×10^4 /well in 24-well plates with DMEM supplemented with 10% calf serum. At 24 h after plating, the cells were incubated for 24 and 48 h in DMEM containing 0–10 μmol/L of OL. (**C**) B16F10 cells were plated at a density of 2.5×10^4 /well in 24-well plates with DMEM supplemented with 10% FBS. Twenty-four hours after plating, the monolayers were serum-starved for 12 h in DMEM. After serum starvation, the cells were incubated for 0–24 h in DMEM containing 0–10 μmol/L of OL. (B, C) Cell viability was estimated using the MTT assay. Each bar represents the mean ± SEM (*n* = 6). Means without a common letter were significantly different, $P < 0.05$. (**D**) 3T3-L1 adipocytes were cultured under normoxic (5% CO_2, 95% air) or hypoxic (1% O_2, 5% CO_2, 94% N_2) conditions for 8 h. Then, conditioned media (CM) were collected. B16F10 cells were plated at a density of 1×10^4 /well in 96-well plates with DMEM supplemented with 10% FBS. At 24 h after plating, the monolayers were serum-starved in DMEM for 2 h. After serum starvation, the cells were treated with the CM for 12 h. BrdU was then added, and the incubation was continued for a further 3 h to analyze BrdU incorporation into DNA (*n* = 5). *Significantly different from the DMEM group; #significantly different from the 3T3-L1-CM in normoxia group, $P < 0.05$.

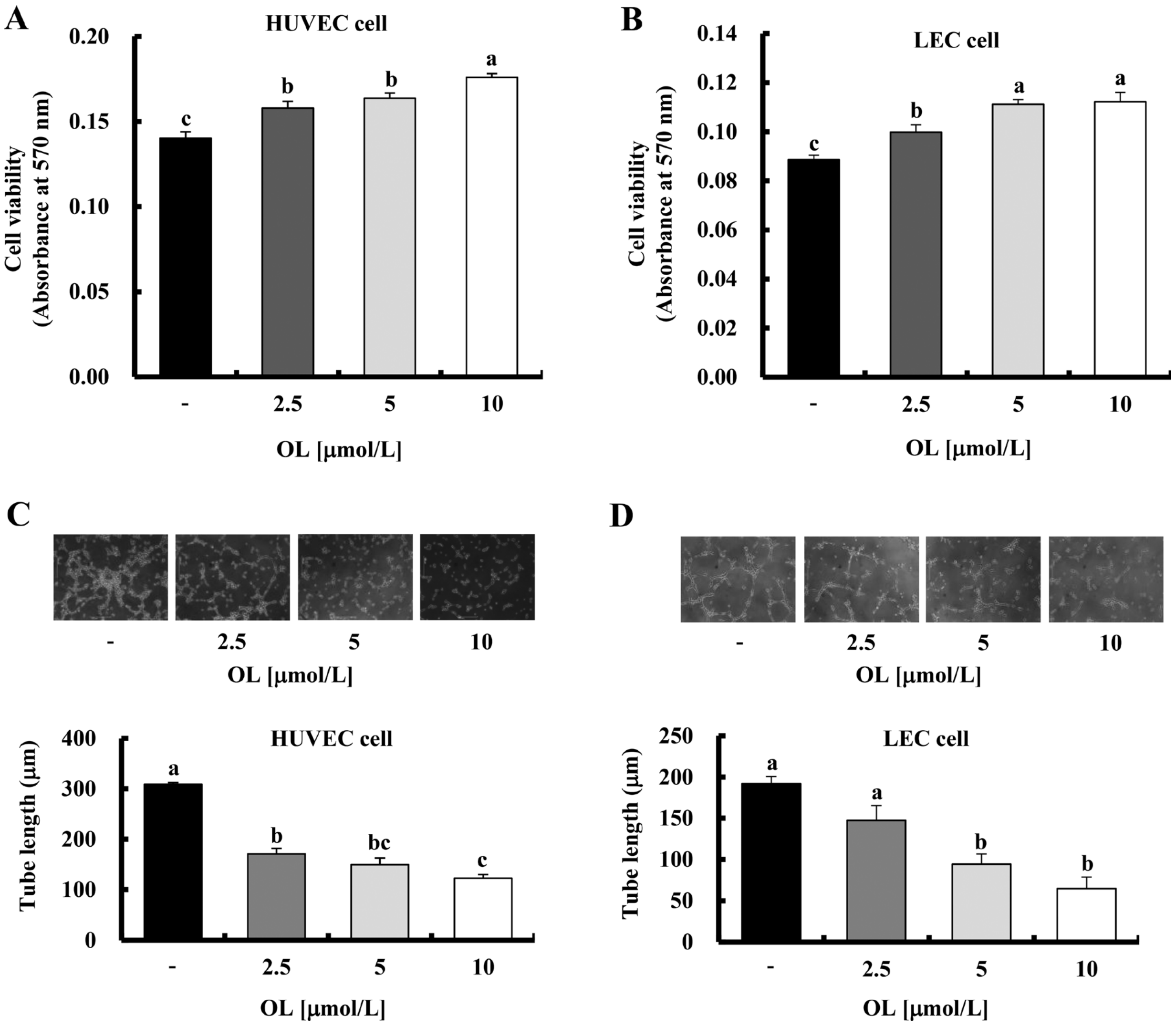

Supplementary Figure 4: OL inhibits tube formation by HUVECs and LECs. (**A**) HUVECs were plated at a density of 5×10^4 /well in 24-well plates with EGM supplemented with 10% FBS. At 24 h after plating, the monolayers were serum-starved in EBM for 2 h. After serum starvation, the cells were incubated for 4 h in EBM containing 0–10 μmol/L of OL. (**B**) LECs were plated at a density of 4×10^4 /well in 24-well plates with EGM supplemented with 5% FBS. At 24 h after plating, the monolayers were serum-starved for 4 h in EBM. After serum starvation, cells were incubated for 4 h in EBM containing 0–10 μmol/L of OL. (A, B) Cell viability was estimated by using MTT assay. Each bar represents the mean ± SEM ($n = 6$). (**C**) HUVECs and (**D**) LECs were plated in Matrigel-coated 24-well plates with 0–10 μmol/L of OL. After 4 h, photographs of tube formation were taken. Total length of formed tubes was measured using the Motic Images Advanced 3.2 system. Each bar represents the mean ± SEM ($n = 3$). Means without a common letter were significantly different, $P < 0.05$.

Supplementary Table 1: Composition of the experimental diets

	Control diet (10 kcal % fat)		High-fat diet (60 kcal % fat)	
	(g)	(kcal)	(g)	(kcal)
**Casein, 80 Mesh**	200	800	200	800
**L-Cystine**	3	12	3	12
**Corn starch**	315	1,260	0	0
**Maltodextrin 10**	35	140	125	500
**Sucrose**	350	1,400	68.8	275.2
**Cellulose**	50	0	50	0
**Soybean oil**	25	225	25	225
**Lard**	20	180	245	2,205
**Mineral mix**	10	0	10	0
**Dicalcium phosphate**	13	0	13	0
**Calcium carbonate**	5.5	0	5.5	0
**Potassium citrate, 1H_2O**	16.5	0	16.5	0
**Vitamin mix**	10	40	10	40
**Choline bitartrate**	2	0	2	0
**Total**	1,055.05	4,057	773.85	4,057

Supplementary Table 2: Primer sequences used in real-time RT-PCR

mRNA		Primer sequences	Reference
CD31	Forward	5′- AGCAAGAAGCAGGAAGGACA -3′	[1]
	Reverse	5′- CATGGGGCAAGGAAGACTC -3′	
F4/80	Forward	5′- CTTTGGCTATGGGCTTCCAGTC -3′	[2]
	Reverse	5′- GCAAGGAGGACAGAGTTTATCGTG -3′	
LYVE-1	Forward	5′- AAGTTCAAAGCCTATTGCCACAA -3′	[3]
	Reverse	5′- GTAAAATGTGGTAACGATTTCTGGAA -3′	
MMR	Forward	5′- CTCGTGGATCTCCGTGACAC -3′	[4]
	Reverse	5′- GCAAATGGAGCCGTCTGTGC -3′	
VEGF-A	Forward	5′- TGTACCTCCACCATGCCAAGT -3′	[1]
	Reverse	5′- TGGAAGATGTCCACCAGGGT -3′	
VEGF-C	Forward	5′- GAAGAAGTTCCACCATCAAACATG -3′	[3]
	Reverse	5′- GACAGTCCTGGATCACAATGCTT -3′	
GAPDH	Forward	5′- CGTGTTCCTACCCCCAATGT -3′	[5]
	Reverse	5′- TGCTTCACCACCTTCTTGATGT -3′	

REFERENCES

1. Chang LK, Garcia-Cardena G, Farnebo F, Fannon M, Chen EJ, Butterfield C, Moses MA, Mulligan RC, Folkman J, Kaipainen A. Dose-dependent response of FGF-2 for lymphangiogenesis. Proceedings of the National Academy of Sciences of the United States of America. 2004; 101:11658–11663.
2. Shaul ME, Bennett G, Strissel KJ, Greenberg AS, Obin MS. Dynamic, M2-like remodeling phenotypes of CD11c+ adipose tissue macrophages during high-fat diet—induced obesity in mice. Diabetes. 2010; 59:1171–1181.
3. Brown HM, Robker RL, Russell DL. Development and hormonal regulation of the ovarian lymphatic vasculature. Endocrinology. 2010; 151:5446–5455.
4. Zhang B, Wang J, Gao J, Guo Y, Chen X, Wang B, Gao J, Rao Z, Chen Z. Alternatively activated RAW264.7 macrophages enhance tumor lymphangiogenesis in mouse lung adenocarcinoma. Journal of cellular biochemistry. 2009; 107:134–143.
5. Sever D, Eldor R, Sadoun G, Amior L, Dubois D, Boitard C, Aflalo C, Melloul D. Evaluation of impaired beta-cell function in nonobese-diabetic (NOD) mouse model using bioluminescence imaging. FASEB journal. 2011; 25:676–684.
